# The Differences Between Intraoperative- and Postoperative-Preferred Music Effects on Emergence Delirium in Elderly Patients: A Single-Center, Prospective Randomized Controlled Trial

**DOI:** 10.3390/medicina61091586

**Published:** 2025-09-02

**Authors:** Hayoung Lee, Eunsung Park, Byoungryun Kim, Cheol Lee

**Affiliations:** 1 Department of Anesthesiology and Pain Medicine, Wonkwang University School of Medicine Hospital, 895 Muwang-ro, Iksan-si 54538, Jeonbuk, Republic of Korea; julia7071@naver.com; 2 Department of Neurosurgery, Wonkwang University School of Medicine Hospital, 895 Muwang-ro, Iksan-si 54538, Jeonbuk, Republic of Korea; silverstar0401@gmail.com; 3 Department of Obstetrics and Gynecology, Wonkwang University School of Medicine Hospital, 895 Muwang-ro, Iksan-si 54538, Jeonbuk, Republic of Korea

**Keywords:** Emergence Delirium, music intervention, general anesthesia, preoperative anxiety

## Abstract

*Background and Objectives*: This study aimed to compare the effects of patient-preferred music delivered intraoperatively versus postoperatively on Emergence Delirium (ED) incidence, severity, and duration, while identifying predictors, to evaluate non-pharmacologic interventions for enhanced anesthetic management. *Materials and Methods*: In a prospective, single-blind, randomized controlled trial, 360 patients aged ≥ 65 years undergoing elective surgery under general anesthesia were randomized to intraoperative music, postoperative music, or control groups. Participants selected genres played via headphones. Primary outcome was ED incidence (Richmond Agitation–Sedation Scale [RASS] score ≥ +1 within 60 min after extubation); secondary outcomes included severity, duration, visual analogue scale pain scores, satisfaction, and adverse events. *Results*: Intraoperative music reduced ED incidence (13.8% vs. 28.7% in controls, *p* < 0.001) and severity (mean RASS 1.3 vs. 1.8, *p* < 0.01). Postoperative music shortened duration (15.2 vs. 22.5 min, *p* < 0.01) and pain (mean visual analogue scale 3.0 vs. 4.2, *p* < 0.01). Both improved satisfaction (*p* < 0.001). Higher preoperative State–Trait Anxiety Inventory scores predicted ED (odds ratio [OR] 1.06, *p* = 0.01), with music protective (OR 0.45–0.62). *Conclusions*: Intraoperative music effectively prevents ED, and postoperative music improves recovery. Integrating patient-preferred music and screening for anxiety may enhance peri-anesthesia care in elderly patients.

## 1. Introduction

Emergence Delirium (ED) is characterized by confusion, restlessness, and agitation as patients awaken from general anesthesia. In elderly patients, ED occurs with a notably high incidence, ranging from 20% to 40%, contributing to longer hospital stays, higher complication rates, and greater demands on anesthesiology staff in the post-anesthesia care unit (PACU) [1]. Symptoms, including hallucinations and hyperexcitability typically emerge 15–30 min post-extubation, endangering patients and providers [2]. Among the known predictors, preoperative anxiety—often measured by the State–Trait Anxiety Inventory (STAI-S)—stands out [3,4], although other factors such as patient age, type of surgery, and anesthetic technique also play roles. Pharmacologic interventions (e.g., benzodiazepines, opioids) carry the risk of adverse effects like respiratory depression, prompting non-pharmacologic alternatives such as music intervention [5]. Music offers a promising, non-pharmacological approach. By engaging emotional and autonomic pathways, music can lower stress, decrease pain perception, and promote relaxation [6,7].

When delivered intraoperatively, music may help stabilize hemodynamic and neurologic responses, potentially mitigating the onset of ED, while postoperative music, played for a designated period after extubation, may alleviate the psychological and sensory distress that contributes to delirium [8,9]. Utilizing patient-preferred selections further enhances these effects by tapping into positive associations and emotional familiarity [7]. Pediatric studies demonstrate intraoperative music’s reduction of ED and pain, suggesting potential benefits for elderly patients [10]. Although pediatric studies have shown that intraoperative music can reduce ED and pain [10], and other trials have explored postoperative benefits, no study has directly compared intraoperative- to postoperative-preferred music in the elderly. Without such guidance, anesthesiologists lack clear protocols for timing and patient selection when using music as a preventive or therapeutic tool in anesthetic management [11].

We hypothesized that intraoperative music would most effectively reduce the incidence and severity of ED by maintaining central nervous system stability, whereas postoperative music would more effectively shorten the duration of delirium and improve recovery metrics. This study, therefore, aimed to compare the effects of preferred music delivered intraoperatively versus postoperatively on the incidence, severity, and duration of ED in older adults. We also examined secondary outcomes—including postoperative pain, patient satisfaction, and adverse events—and employed multivariable logistic regression to identify predictors of ED.

## 2. Materials and Methods

### 2.1. Study Design and Participants

This prospective, single-blind, randomized controlled trial was approved by the Institutional Review Board of Wonkwang University Hospital (IRB No. WKUH 2025-04-021-0045). Written informed consent was obtained from all participants before randomization. The study followed the 2013 Declaration of Helsinki and was registered on ClinicalTrials.gov (NCT 06970249). We enrolled adults aged 65 or older with American Society of Anesthesiologists (ASA) physical status I–III who were scheduled for elective surgery lasting from 1 to 3 h under general anesthesia. Surgical types included orthopedic (e.g., joint replacements, fracture repairs; 33%) and other procedures (general surgery such as laparoscopic cholecystectomy or hernia repair [45%], urologic such as transurethral resection [15%], and gynecologic such as hysterectomy [7%]) in Supplementary Table S1 for detailed surgical types. All participants had a Mini-Mental State Examination (MMSE) score of 20 or higher and no significant hearing impairment. We excluded patients undergoing emergency procedures, those with severe psychiatric illness, individuals with an MMSE below 20, or any condition that would prevent informed consent.

### 2.2. Randomization and Blinding

Using Stata 17.0 (StataCorp, College Station, TX, USA), a computer-generated randomization sequence was created using block randomization with variable block sizes of 3 and 6 to maintain allocation concealment. Randomization was stratified by three prognostic factors: age group (65–75 vs. >75 years), sex (male vs. female), and surgery type (orthopedic vs. other). Eligible participants were randomly assigned in a 1:1:1 ratio to one of three groups: intraoperative music, postoperative music, or control. Group assignments were placed in sequentially numbered, sealed, opaque envelopes by an independent researcher not involved in patient recruitment or outcome assessment. After obtaining informed consent, a research assistant opened the next envelope in sequence to determine group allocation. Blinding was maintained for all outcome assessors, including PACU nurses, who were unaware of group assignments. Although anesthesiologists were aware of group assignments, outcome assessors in the PACU remained blinded to minimize detection bias. Although anesthesiologists and PACU staff administered the music intervention, they were not involved in data collection or statistical analysis to minimize bias.

### 2.3. Interventions

No benzodiazepine premedication was given. General anesthesia was induced with propofol (2 mg/kg), maintained with sevoflurane, and supplemented with remifentanil (0.05–0.2 µg/kg/min). Rocuronium (0.6–0.8 mg/kg) was administered for intubation with additional 0.1–0.2 mg/kg boluses guided by train-of-four (TOF) monitoring to maintain 1–2 twitches during surgery. Reversal was carried out at case end with sugammadex 2 mg/kg (or pyridostigmine 0.2 mg/kg with glycopyrrolate 10 µg/kg if sugammadex was unavailable) targeting the TOF ratio ≥ 0.9 prior to extubation. Intraoperative ventilation used a volume-controlled mode with tidal volume of 6–8 mL/kg predicted body weight, PEEP 5 cmH_2_O, I:E ≈ 1:2, FiO_2_ 0.4–0.5 (titrated to SpO_2_ ≥ 95%), and EtCO_2_ maintained at 35–40 mmHg by adjusting the respiratory rate. Airway management was via cuffed orotracheal intubation following induction. Cuff pressure was maintained at 20–25 cmH_2_O and verified after positioning. Extubation followed standard criteria (adequate spontaneous ventilation, TOF ratio ≥ 0.9, and command following). Anesthesia depth was monitored via BIS (40–60), minimizing the risk of awareness. Intraoperative ventilation used the volume-controlled mode with a tidal volume 6–8 mL/kg predicted body weight, PEEP 5 cmH_2_O, I:E ≈ 1:2, FiO_2_ 0.4–0.5 (titrated to SpO_2_ ≥ 95%), and EtCO_2_ maintained at 35–40 mmHg by adjusting respiratory rate. Patient-preferred music (e.g., classical, jazz, ambient) was selected preoperatively from a standardized list and played via headphones at 40–60 dB, as measured by a sound meter. Although participants chose their preferred genre, all tracks were matched for tempo (60–80 bpm) and volume (40–60 dB) to reduce variability. Music was played from induction to extubation; postoperatively, music was offered for 60 min after extubation in the PACU. The control group received standard care with ambient sounds. Intraoperative hypotension (mean arterial pressure < 20% of baseline) was managed with ephedrine (5–10 mg), and postoperative pain was treated with fentanyl (0.5 µg/kg) as needed.

### 2.4. Outcome Measures

The primary outcome was the incidence of ED, defined as a Richmond Agitation–Sedation Scale (RASS) score ≥ +1 within 60 min after extubation, assessed every 15 min by two trained PACU staff (inter-rater κ = 0.85). RASS was chosen for its PACU applicability and correlation with delirium signs during emergence, though CAM may complement it in future work. Secondary outcomes included the severity of ED (highest RASS score within 60 min), duration of ED (minutes from extubation to RASS ≤ 0), postoperative pain measured by the visual analogue scale, patient satisfaction (5-point Likert scale, 1 = very dissatisfied, 5 = very satisfied, at PACU discharge), adverse events (e.g., incidence of nausea, vomiting, respiratory depression, and other complications), and preoperative anxiety (STAI-S, 20–80, with 10 positively worded items reverse-scored). Adverse events (nausea/vomiting) were prospectively assessed within 60 min after extubation in the PACU at the same 15 min intervals. VAS pain scores (0–10) were obtained every 15 min for 60 min post-extubation, only from responsive patients (RASS ≤ 0); delirious episodes were managed first. Preoperative anxiety was measured using the STAI-S on the day before the operation; scores ranged from 20 to 80, with higher scores indicating greater anxiety (10 reverse-scored items). All data were captured on a standardized Case Report Form, including demographics, MMSE, anesthetic details, and postoperative outcomes.

### 2.5. Sample Size and Statistical Analysis

The 30% baseline ED incidence and 15% expected reduction were based on prior meta-analyses and preliminary institutional data [5,11]. We calculated that 108 patients per arm would provide 80% power to detect a reduction in ED incidence from 30% (control) to 15% (music), at α = 0.05 (Cohen’s h = 0.36). Allowing for a 10% dropout, we enrolled 360 patients (120 per group). The sample size was powered for the primary outcome only. Secondary outcome analyses are exploratory. Analyses were performed on an intention-to-treat (ITT) basis; a per-protocol (PP) analysis included only those who fully adhered to their assigned intervention. Continuous variables (e.g., RASS, VAS, satisfaction) were assessed for normality using the Shapiro–Wilk test. Normally distributed data were compared using ANOVA with Tukey’s post hoc correction (adjusted α = 0.01 for four secondary comparisons). Non-normal data (e.g., ED duration) were log-transformed or analyzed using the Kruskal–Wallis test if the transformation failed. Categorical outcomes (e.g., ED incidence) were compared using χ^2^ tests.

We used multivariable logistic regression to identify predictors of ED, adjusting for age, sex, BMI, MMSE, preoperative STAI-S, surgery type, and music group, including the music × STAI-S interaction term. Interaction analysis between preoperative anxiety and intervention group was preplanned based on previous evidence linking anxiety to ED. Missing data (<3% of STAI-S or RASS items) were imputed using multiple imputation with chained equations (MICE) to preserve variance, while sensitivity analyses used only complete cases. Subgroup analyses examined effects by age group, sex, and surgery type. All secondary pairwise comparisons were corrected with Bonferroni adjustment to control for type I error. All tests were two-tailed; *p* < 0.05 indicated statistical significance for the primary outcome and regression models, with adjusted *p*-values reported for secondary outcomes. Statistical analyses were performed using SPSS 29 (IBM Corp., Armonk, NY, USA). Analyses followed a prespecified SAP finalized before unblinding. The primary model adjusted for randomization stratification factors (age, sex, surgery type). A prespecified sensitivity model further adjusted for BMI, MMSE, and preoperative STAI-S as prognostic covariates.

## 3. Results

### 3.1. Baseline Characteristics

**Figure 1:**
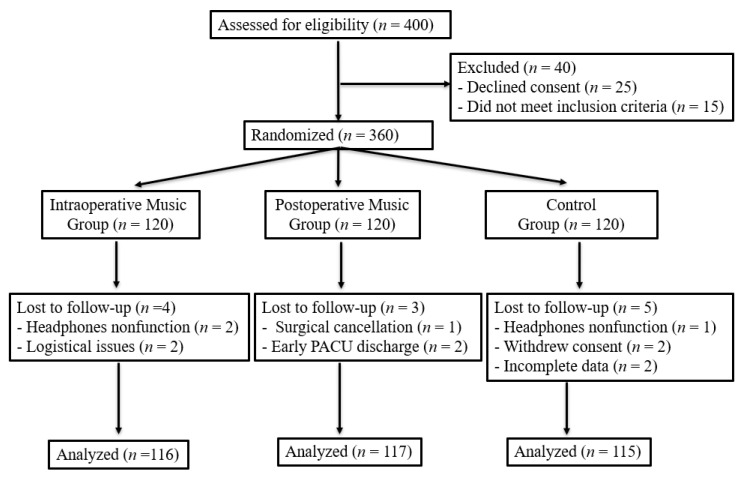
CONSORT flow diagram.

We assessed 400 patients for eligibility and randomized 360 into three groups (120 intraoperative music, 120 postoperative music, and 120 control); 40 were excluded (25 declined consent, 15 did not meet inclusion criteria). After randomization, 4 patients in the intraoperative music group, 3 in the postoperative music group, and 5 in the control group were lost to follow-up, leaving 116, 117, and 115 patients, respectively, for analysis (total analyzed = 348) (Figure 1).

Baseline characteristics did not differ among groups (Table 1; *p* > 0.05). The overall mean age was 72.4 ± 5.8 years; 58% of the participants were female. The MMSE score averaged 25.6 ± 2.7, and preoperative STAI-S scores indicated moderate anxiety (43.2 ± 9.1). Preferred music genres were 50% classical, 30% ambient, 15% jazz, and 5% other. No data were missing for the primary outcome; fewer than 3% of STAI-S items were imputed (Table 1).

#### 3.1.1. Primary Outcome: Incidence and Severity of Emergence Delirium

The incidence of ED was lower with intraoperative music (13.8%, 16/116; risk ratio = 0.48, 95% CI [0.28, 0.81]) compared with the control (28.7%, 33/115; *p* = 0.00) and with postoperative music (18.8%, 22/117; *p* = 0.04). Intraoperative music also reduced the severity of ED, as reflected by the highest RASS score (1.3 ± 0.5, 95% CI [1.1, 1.5]) versus the control (1.8 ± 0.7, 95% CI [1.6, 2.0]; *p* = 0.00, adjusted *p* = 0.00) and versus postoperative music (1.5 ± 0.6, 95% CI [1.3, 1.7]; *p* = 0.03, adjusted *p* = 0.03) (Table 2).

#### 3.1.2. Secondary Outcomes: Duration of Emergence Delirium, Pain, and Satisfaction

The duration of ED was shortest in the postoperative music group (15.2 ± 6.1 min, 95% CI [13.8, 16.6]) compared with the control (22.5 ± 8.3 min, 95% CI [20.7, 24.3]; *p* = 0.00, adjusted *p* = 0.00) and intraoperative music (18.4 ± 7.0 min, 95% CI [16.8, 20.0]; *p* = 0.02, adjusted *p* = 0.02).

After extubation, postoperative pain (VAS) was lower with postoperative music (3.0 ± 1.4, 95% CI [2.7, 3.3]) than with the control (4.2 ± 1.7, 95% CI [3.8, 4.6]; *p* = 0.00, adjusted *p* = 0.00) and intraoperative music (3.5 ± 1.5, 95% CI [3.2, 3.8]; *p* = 0.03, adjusted *p* = 0.03). Patient satisfaction at PACU discharge was higher in both music groups (intraoperative: 4.2 ± 0.8, 95% CI [4.0, 4.4]; postoperative: 4.4 ± 0.7, 95% CI [4.2, 4.6]) than in controls (3.6 ± 0.9, 95% CI [3.4, 3.8]; *p* = 0.00, adjusted *p* = 0.00) (Table 2).

#### 3.1.3. Hemodynamic Parameters and Adverse Events

Hemodynamic parameters remained stable across groups (Table 2; *p* > 0.05), with MAP at 60 min after extubation of 84.8 ± 9.5 mmHg (intraoperative), 85.3 ± 9.2 mmHg (postoperative), and 84.1 ± 10.1 mmHg (control), and HR of 71.2 ± 8.3, 72.0 ± 8.7, and 73.1 ± 9.0 bpm, respectively. Adverse events (postoperative nausea/vomiting) occurred in 4.3% of participants (15/348: 5 intraoperative, 4 postoperative, and 6 control; *p* = 0.81). Fentanyl use (0.5 µg/kg) after extubation was less frequent in the postoperative music group (12%, 14/117) than in controls (22.6%, 26/115; *p* = 0.02).

### 3.2. Predictors and Subgroup Analysis

In multivariable logistic regression, higher preoperative STAI-S scores were associated with a greater risk of ED (OR = 1.06, 95% CI [1.02, 1.10], *p* = 0.01). Intraoperative music reduced the odds of ED (OR = 0.45, 95% CI [0.28, 0.72], *p* = 0.00), as did postoperative music (OR = 0.62, 95% CI [0.40, 0.95], *p* = 0.03), and there was a significant music × STAI-S interaction (OR = 0.90, 95% CI [0.84, 0.97], *p* = 0.03). Age, sex, BMI, MMSE, and surgery type did not predict ED (all *p* > 0.05). A subgroup analysis showed that the benefit of intraoperative music was especially pronounced in patients older than 75 years *p* = 0.02) (Table 3).

### 3.3. Sensitivity Analysis

Effect estimates were consistent in the prespecified sensitivity model that additionally adjusted for BMI, MMSE, and preoperative STAI-S (Table S1), supporting the robustness of the primary findings.

## 4. Discussion

The primary finding of this study is that intraoperative-preferred music significantly lowered the incidence of ED (13.8% vs. 28.7% in controls; *p* < 0.001; RR = 0.48, 95% CI [0.28, 0.81]) and the severity of ED (mean highest RASS 1.3 ± 0.5 vs. 1.8 ± 0.7; *p* < 0.01). This effect may relate to music’s ability to balance neurological and autonomic responses during anesthesia by influencing subconscious auditory processing, lowering cortisol levels through reduced activation of the hypothalamic–pituitary–adrenal axis, and stabilizing EEG patterns to prevent hyperarousal during emergence, thereby reducing postoperative agitation [6,10]. Our logistic regression confirmed that patients who listened to intraoperative music were less likely to develop ED (OR = 0.45, *p* < 0.001). Additionally, this protective effect was especially notable among those with higher preoperative anxiety (music × STAI-S interaction, OR = 0.90; *p* = 0.03). Selecting patient-preferred playlists likely increased emotional involvement and helped produce these benefits [7].

The observed decrease in both the incidence and severity of ED has significant implications for anesthesiology practice. Emergence Delirium not only raises the risk of patient harm and extends PACU stays but also adds to the workload of anesthesiology staff [12]. By incorporating intraoperative music, anesthesiologists have a simple, non-pharmacologic method to improve patient safety and facilitate recovery, especially for those identified as high risk through STAI-S screening [3]. Unlike sedatives or opioids, music carries no risk of respiratory depression and can help achieve faster, safer transitions through the PACU [13]. The practicality of a headphone-delivered intervention makes it easy to implement in busy clinical settings and supports holistic, patient-centered care models in anesthesiology [14,15,16].

Postoperative music administered for 60 min after extubation significantly shortened the duration of ED (15.2 ± 6.1 vs. 22.5 ± 8.3 min; *p* < 0.01) and reduced pain intensity (mean VAS 3.0 ± 1.4 vs. 4.2 ± 1.7; *p* < 0.01). Although statistically significant, the 7 min reduction in ED duration may have limited clinical relevance depending on PACU workflow. These benefits likely reflect music’s ability to distract from discomfort and activate endogenous analgesic pathways, such as the release of endorphins and dopamine in reward centers, while consciously engaging cognitive distraction and rhythmic entrainment to lower agitation levels through enhanced neural synchronization [7]. Furthermore, the postoperative music group required fewer opioids (12% vs. 22.6%; *p* = 0.02), which may further reduce opioid-related side effects in elderly patients [9]. Both music groups reported higher satisfaction scores at discharge from the PACU, highlighting the positive impact of music on patient experience.

Our findings build on previous research by those who showed reduced agitation with preferred music in patients with traumatic brain injury [7] and by those who found that intraoperative music decreased ED and pain in a pediatric group [10]. Unlike studies using non-personalized music, our use of patient-preferred selections likely enhanced emotional engagement and treatment effectiveness. To our knowledge, this is the first trial comparing intraoperative- to postoperative-preferred music in an elderly surgical population and the first to evaluate anxiety–music interactions as predictors of ED [11]. We did not consider other potential risk factors for ED, such as sleep deprivation, a past history of delirium, or intraoperative auditory stimulation from environmental noise.

This study has some limitations. First, the single center in Korea limits how well our findings can be applied to other cultural contexts, as music preferences and their effects can differ across cultures [17]. Because music preference and emotional responses vary by culture, multicenter trials across different regions are necessary to confirm our results. Second, while patient-preferred music improves effectiveness, it introduces variability in genre and tempo that we could not fully control. Third, although our reliance on RASS scores showed high inter-rater reliability (*κ* = 0.85), observer bias may still affect results. Using objective measures, such as EEG, in future studies could improve assessment accuracy [18]. Fourth, we did not assess auditory evoked potentials, which could confirm subconscious processing, and subconscious auditory processing likely mediated effects, but future studies with AEP monitoring could refine this understanding. Fifth, RASS focuses on agitation; integrating CAM could enhance specificity, though its post-extubation feasibility is limited. Sixth, VAS reliability during recovery may be affected by residual agitation; non-verbal pain tools (e.g., behavioral scales) could be explored in future studies. Finally, although the study was adequately powered to identify differences in ED incidence, it was underpowered for some secondary outcomes (e.g., pain), and validation in multicenter settings will be essential.

## 5. Conclusions

Intraoperative-preferred music reduces the incidence and severity of ED, while postoperative music shortens its duration and alleviates pain in elderly surgical patients. High preoperative anxiety predicts ED, with music providing a protective effect, particularly during surgery. These findings provide anesthesiologists with non-pharmacologic tools to enhance safety and recovery. Further research should investigate multicenter settings, objective measures of ED, and standardized music protocols to confirm efficacy and optimize geriatric care in anesthesiology.

## Figures and Tables

**Table 1:** Baseline patient characteristics.

Variable	Intraoperative Music Group (*n* = 116)	Postoperative Music Group (*n* = 117)	Control Group (*n* = 115)	*p*-Value
Age (years)	72.1 ± 5.7	72.6 ± 6.0	72.5 ± 5.8	0.88
Sex (F/M)	68/48	66/51	68/47	0.92
BMI (kg/m^2^)	23.8 ± 3.4	24.0 ± 3.6	23.7 ± 3.5	0.81
ASA (I/II/III)	29/58/29	31/57/29	28/58/29	0.97
Surgery type				0.91
Orthopedic surgery	39 (33.6)	37 (31.6)	35 (30.4)	
Other	77 (66.4)	80 (68.4)	80 (69.6)	
MMSE Score	25.7 ± 2.6	25.5 ± 2.8	25.6 ± 2.7	0.89
STAI-S Score	43.4 ± 9.0	43.0 ± 9.2	43.2 ± 9.1	0.94
Preferred music genre				0.99
Classical	58 (50.0)	59 (50.4)	57 (49.6)	
Ambient	35 (30.2)	35 (29.9)	34 (29.6)	
Jazz	17 (14.7)	18 (15.4)	17 (14.8)	
Other	6 (5.2)	5 (4.3)	7 (6.1)	

Notes: Data are presented as mean ± SD or *n* (%). BMI, Body Mass Index; ASA, American Society of Anesthesiologists; MMSE, Mini-Mental State Examination; STAI-S, State–Trait Anxiety Inventory.

**Table 2:** Primary and secondary outcomes.

Outcome	Intraoperative Music Group (*n* = 120)	Postoperative Music Group (*n* = 120)	Control Group (*n* = 120)	*p*-Value (Adjusted *p*-Value)
ED Incidence (RASS ≥ +1)	16 (13.8%) *	22 (18.8%) *	33 (28.7%)	0.00 (N/A)
ED Severity (RASS)	1.3 ± 0.5 *	1.5 ± 0.6	1.8 ± 0.7	0.00 (0.00)
ED Duration (min)	18.4 ± 7.0	15.2 ± 6.1 *^†^	22.5 ± 8.3	0.00 (0.00)
Pain Score (VAS, 0–10)	3.5 ± 1.5	3.0 ± 1.4 *^†^	4.2 ± 1.7	0.00 (0.00)
Satisfaction (1–5)	4.2 ± 0.8 *	4.4 ± 0.7 *	3.6 ± 0.9	0.00 (0.00)
MAP (mmHg, PACU 60 min)	84.8 ± 9.5	85.3 ± 9.2	84.1 ± 10.1	0.72 (0.72)
HR (bpm, PACU 60 min)	71.2 ± 8.3	72.0 ± 8.7	73.1 ± 9.0	0.65 (0.65)
Adverse Events (Postoperative Nausea/Vomiting)	5 (4.3%)	4 (3.4%)	6 (5.2%)	0.81 (N/A)
Fentanyl Use (0.5 µg/kg)	16 (13.8%) *	14 (12.0%) *	26 (22.6%)	0.02 (N/A)

Notes: Data are presented as mean ± SD or *n* (%). ED, Emergence Delirium; RASS, Richmond Agitation–Sedation Scale; VAS, Visual Analog Scale; MAP, Mean Arterial Pressure; HR, Heart Rate. * *p* < 0.05 vs. control; ^†^ *p* < 0.05 vs. intraoperative.

**Table 3:** Logistic regression analysis of predicting factors for Emergence Delirium.

Variable	OR (95% CI)	*p*-Value
Age (years)	1.02 (0.98, 1.06)	0.31
Sex (Female vs. Male)	1.15 (0.78, 1.70)	0.48
BMI (kg/m^2^)	0.99 (0.93, 1.05)	0.71
MMSE Score	0.97 (0.90, 1.04)	0.39
Preoperative STAI-S Score	1.06 (1.02, 1.10) *	0.01
Orthopedic	1.12 (0.72, 1.74)	0.62
Other	0.95 (0.58, 1.56)	0.84
Intraoperative Music	0.45 (0.28, 0.72) *	0.00
Postoperative Music	0.62 (0.40, 0.95) *	0.03
Music × STAI-S Interaction	0.90 (0.84, 0.97) *	0.03

Notes: Data are presented as odds ratios (OR) with 95% confidence intervals (CI). STAI-S, State–Trait Anxiety Inventory; MMSE, Mini-Mental State Examination; BMI, Body Mass Index. * *p* < 0.05.

## Data Availability

Anonymized data supporting this study’s findings are available from the corresponding author upon reasonable request.

## Supplementary Materials

The following supporting information can be downloaded at https://www.mdpi.com/article/10.3390/medicina61091586/s1, Table S1 for detailed surgical types.

## Abbreviations

ED	Emergence Delirium
RASS	Richmond Agitation–Sedation Scale
VAS	Visual Analogue Scale
STAI-S	State–Trait Anxiety Inventory-State
PACU	Post-Anesthesia Care Unit
ASA	American Society of Anesthesiologists
MMSE	Mini-Mental State Examination
BIS	Bispectral Index
IRB	Institutional Review Board
ITT	Intention To Treat
PP	Per-Protocol
ANOVA	Analysis of Variance
BMI	Body Mass Index
MAP	Mean Arterial Pressure
HR	Heart Rate
OR	Odds Ratio
CI	Confidence Interval
RR	Risk Ratio
EEG	Electroencephalogram
dB	Decibels
