# Supplementary material for: The Differences Between Intraoperative- and Postoperative-Preferred Music Effects on Emergence Delirium in Elderly Patients: A Single-Center, Prospective Randomized Controlled Trial
Source: Medicina (Kaunas). 2025 Sep 2;61(9):1586. doi: 10.3390/medicina61091586 (PMC12471603; doi:10.3390/medicina61091586)
Supplement: Supplementary file 1 [file medicina-61-01586-s001.zip › medicina-3825647-supplementary.pdf]

Supplementary Table S1 for detailed surgical types.

Surgical Type	Intraoperative Music Group (n=116)	Postoperative Music Group (n=117)	Control Group (n=115)	Total (n=348)
**Orthopedic**	39 (33.6%)	37 (31.6%)	35 (30.4%)	111 (31.9%)
- Joint replacement (knee/hip)	25 (21.6%)	24 (20.5%)	23 (20.0%)	72 (20.7%)
- Fracture repair	14 (12.1%)	13 (11.1%)	12 (10.4%)	39 (11.2%)
**General Surgery**	35 (30.2%)	36 (30.8%)	35 (30.4%)	106 (30.5%)
- Laparoscopic cholecystectomy	18 (15.5%)	19 (16.2%)	18 (15.7%)	55 (15.8%)
- Hernia repair	17 (14.7%)	17 (14.5%)	17 (14.8%)	51 (14.7%)
**Urologic**	23 (19.8%)	23 (19.7%)	23 (20.0%)	69 (19.8%)
- Transurethral resection	23 (19.8%)	23 (19.7%)	23 (20.0%)	69 (19.8%)
**Gynecologic**	19 (16.4%)	21 (17.9%)	22 (19.1%)	62 (17.8%)
- Hysterectomy	19 (16.4%)	21 (17.9%)	22 (19.1%)	62 (17.8%)
